# Attraction, mobility, and preference by *Lasioderma serricorne* (Coleoptera: Ptinidae) to microbially-mediated volatile emissions by two species of fungi in stored grain

**DOI:** 10.1038/s41598-023-32973-y

**Published:** 2023-04-15

**Authors:** Marco A. Ponce, Petra Sierra, Jacqueline M. Maille, Tania N. Kim, Erin D. Scully, William R. Morrison

**Affiliations:** 1 Department of Entomology, Kansas State University, 123 W. Waters Hall, 1603 Old Claflin Place, Manhattan, KS 66506 USA; 2 Biology Department, Kalamazoo College, 1200 Academy St., Kalamazoo, MI 49006 USA; 3 USDA, Agricultural Research Service, Center for Grain and Animal Health Research, 1515 College Ave., Manhattan, KS 66502 USA

**Keywords:** Microbial ecology, Agroecology

## Abstract

Insects and microbes are known to interact in a variety of ways at food facilities, compounding damage. However, little research has explicated how specific common fungal species affect the behavior of the cosmopolitan secondary stored product pest, *Lasioderma serricorne*. Enhanced knowledge about attraction to microbially-produced volatile organic compounds (MVOCs) may be used to manipulate insect behavior. *Aspergillus flavus* and *Fusarium verticillioides* are two common, widespread pre- and postharvest fungi on small cereals that produce aflatoxins and fumonisins, respectively, while directly competing with each other for nutrients. Our goals were to (1) characterize the volatile emissions from grain inoculated by *A. flavus* or *F. verticillioides* derived from the cuticle of *L. serricorne* compared to uninoculated and sanitized grain, and (2) understand how MVOCs from each fungal species affects mobility, attraction, and preference by *L. serricorne*. Headspace collection revealed that the *F. verticillioides*- and *A. flavus*-inoculated grain produced significantly different volatiles compared to sanitized grain or the positive control. Changes in MVOC emissions affected close-range foraging during an Ethovision movement assay, with a greater frequency of entering and spending time in a small zone with kernels inoculated with *A. flavus* compared to other treatments. In the release-recapture assay, MVOCs were found to be attractive to *L. serricorne* at longer distances in commercial pitfall traps. There was no preference shown among semiochemical stimuli in a still-air, four-way olfactometer. Overall, our study suggests that MVOCs are important for close- and long-range orientation of *L. serricorne* during foraging, and that MVOCs may have the potential for inclusion in behaviorally-based tactics for this species.

## Introduction

Insects inflict over $100 billion in economic losses to commodities after harvest^1^, while microbes, especially fungi, may endanger the safety of human through the production of mycotoxins^2,3^. Insects and microbes may interact in a variety of ways at food facilities, compounding damage and threatening food security. Although insects and microbes may be found together in food facilities, they are often not discussed together even though microbes can alter the grain in environment in ways that are beneficial to stored product pests^4^. For example, the presence of microbes increased progeny production of *Sitophilus oryzae* (L.) (Coleoptera: Curculionidae) and simultaneously elevated the humidity and temperature of the grain mass, thereby creating favorable growth conditions for themselves and *S. oryzae*. *Fusarium verticillioides* likewise was found to increase the progeny of *Sitophilus zeamais* (Coleoptera: Curculionidae)^5^. Hubert et al.^6^ found that the abundance of bacteria in spent growth medium was positively correlated with mite fitness.

In addition to altering the abiotic environment, microbes produce microbial volatile organic compounds (MVOCs, hereafter), which may act as insect pheromones or food kairomones that mediate key behaviors including attraction, repulsion, mating, foraging, dispersal, and aggregation^7^. In a recent metanalysis, Ponce et al.^4^ found that insect behavioral responses (e.g., attraction, repellency, or net neutrality) to MVOCs are complex and can vary by microbial taxa and community composition, abiotic environment, insect species, life history (internal-infesting or external-infesting stored product insects). Steiner et al.^8^ found the stored product parasitoid, *Lariophagus distinguendus* Förster (Hymenoptera: Pteromalidae), avoided the fungal volatile 1-octen-3-ol. Van Winkle et al.^9^ found that tempering grain to different moisture levels and incubating for 9–28 days at 30 °C significantly changed the blend of volatiles and microbial communities, which showed attraction at moderate levels to *Rhyzopertha dominica* (F.) (Coleoptera: Bostrichidae), but not the secondary pest, *Tribolium castaneum* (Herbst) (Coleoptera: Tenebrionidae). Ponce et al.^10^ found that MVOCs affected the close-range foraging of an internal-infesting pest, *S. oryzae*, after inoculation of grain with different life stages (e.g., sexual vs. asexual) of *Aspergillus flavus*. An important knowledge gap that needs to be addressed is how and whether fungal MVOCs impact behavior of external-infesting stored product pests.

One important external-infesting pest is the cigarette beetle, *Lasioderma serricorne* (F.) (Coleoptera: Ptinidae). This is a cosmopolitan pest globally in and around grain storage and food processing facilities that has the unique ability to detoxify plant secondary metabolites, including nicotine and caffeine, through interactions with its midgut symbionts^11^. Contrary to its common name, *L. serricorne* has one of the most diverse host breadths behind *Tribolium castaneum* (Herbst) (Coleoptera: Tenebrionidae)^11^, feeding on 222 different dried plant and animal products and reproducing on 49 commodities^12^. The injury inflicted by *L. serricorne* is regularly severe, and includes reduced market value from direct feeding, quality decreases from excrement and exuvia, and loss of business from consumer complaints^11,13^. *Lasioderma serricorne* has been proposed as a new model species to evaluate symbioses with microorganisms^14^, and certainly its interaction with the microbiome of grain pathogens should be included under this umbrella. This species is highly mobile, able to disperse readily by flight during most of the year in Mediterranean climates^15^ and possesses strong walking and climbing capabilities^16^. There have also been promising preliminary reports showing control of *L. serricorne* may be possible with semiochemical-based strategies^17–19^. Prior work in Japan isolated 17 species of molds and 5 species of yeasts from the cuticles of *L. serricorne*, including *Aspergillus*, *Arthrinium*, *Eurotium*, *Fusarium*, and *Penicillium*^20^. However, the ecological role of these microbes and how they affect *L. serricorne* behavior is poorly understood.

Pathogens that colonize raw grain and finished products also threaten commodities and can have significant negative impacts on both human and animal health^21–23^. In addition, off-odors in raw and finished commodities have also been linked to microbial contamination^24^. Common fungal species that contaminate grain and commodities post-harvest typically include those from the genera *Aspergillus*, *Fusarium*, *Penicillium*, and *Alternaria*, among others. However, prior work has frequently found that *Aspergillus* spp. are the most common genera in flour production, with 35% of 1258 samples belonging to this genus from various food facilities in Europe, and a significant portion (10%) belonging to *Fusarium* spp. as well^25^. Likewise, *Aspergillus*, *Penicillium* and *Fusarium* spp. are the main micro-organisms involved in contamination of maize^26^. Specifically, *A. flavus* and *F. verticillioides* were most frequently isolated from Brazilian maize, and have a high proportion of strains that produce aflatoxins and fumonisins^27^. *A. flavus* and *F. verticillioides* are direct competitors on grains, and *A. flavus* is expected to outcompete other *Fusarium* spp. in small cereals under climate change going forward, with documented higher incidence during particularly warm, dry years^28,29^. Thus, in this work, we focus on these two genera, including *A. flavus*, a widespread post-harvest plant pathogen^30^, which causes significant economic losses after harvest^2^ that is known to produce the carcinogenic secondary metabolite, aflatoxin B_1_^31^. Although much progress has been made in reducing aflatoxin contamination, it remains a sporadic problem for corn stored in the southern United States and it is routinely detected in stored commodities at markets in developing countries^32^. The other focus of this study, and a common fungal contaminant of cereals is *Fusarium verticillioides*, which may attack wheat either pre- or post-harvest^33^. Under most years in Italy, contamination of maize with *F. verticillioides* is a concern, which can produce fumonisins^28^. Maize from smallholder farms in Vietnam were found to be predominately contaminated with *F. verticillioides* and fumonisin B_1_, with traditional harvest and postharvest practices correlating to higher contamination^34^. This is more broadly a problem for smallholders around the world, with *A. flavus* and *F. verticillioides* implicated in aflatoxin and fumonisin production, including in Africa^35^. Despite the high frequency of these fungal pathogens in postharvest environments, little work has been done to investigate headspace volatiles of grain contaminated by either microbe and the behavioral impacts on stored product insects associated with commodities.

One alternative suite of tactics in IPM to combat stored product pests are semiochemical-mediated, behaviorally-based tactics (reviewed recently in Morrison et al.^36^) that use volatile cues to manipulate behavior in ways that are conducive to protecting commodities from insect colonization. Effective attractants and/or repellents are required for the success of these IPM tactics. One class of stimuli that may be broadly attractive to stored product insects are MVOCs, partially because of the evolutionary history of these taxa that involved using animal caches of food before the advent of agriculture. One particular application of MVOCs may be in attract-and-kill, whereby insects are attracted to a spatially-circumscribed area by a long-range attractant treated with an insecticide and then removed from the foraging population (e.g.,^37–40^). Recent work has found that attract-and-kill based interception traps placed on the perimeter of food facilities were successful at preventing progeny production when they included long-lasting insecticide netting, capturing over 3000 individuals in weekly 48-h deployments in 2 years^39^. However, a challenge in deploying this tactic at food facilities is competing with existing volatile stimuli, including nearby food resources, insects emitting aggregation or sex pheromones, and sites of spillage. Nonetheless, the incorporation of MVOCs into lures for attract-and-kill tactics may make these volatile sources unique among the backdrop of odors at food facilities and support delivering improved and unique IPM tools in the food environment to stakeholders.

Thus, our goals were to (1) characterize the volatile emissions from grain inoculated by *Aspergillus flavus* or *Fusarium verticillioides* derived from the cuticle of *L. serricorne* compared to uninoculated and sanitized grain, and (2) understand how MVOCs from each fungal species affects mobility, attraction, and preference by *L. serricorne*. We hypothesized that headspace blends will be unique among our fungal treatments, and that this will lead to differences in preference and mobility by *L. serricorne*.

## Results

### Fungal morphotype sequencing

After running a BLASTn search against the nt database, the top ten matches for the ITS consensus sequences from the AF morphotype were all with isolates of *Aspergillus*, with nine of them *A. flavus* and one of them *A. niger* with 99% sequence identity and 97–98% query coverage (E ≈ 0). Among the top 10 matches for the ITS sequence of the FS morphotype, 90% were to *F. verticillioides*, while one match was to *Fusarium annulatum*. There was 99% sequence identity with the matches over 43% query coverage (E ≈ 0) for the FS morphotype.

### Four-way olfactometer

**Figure 1:**
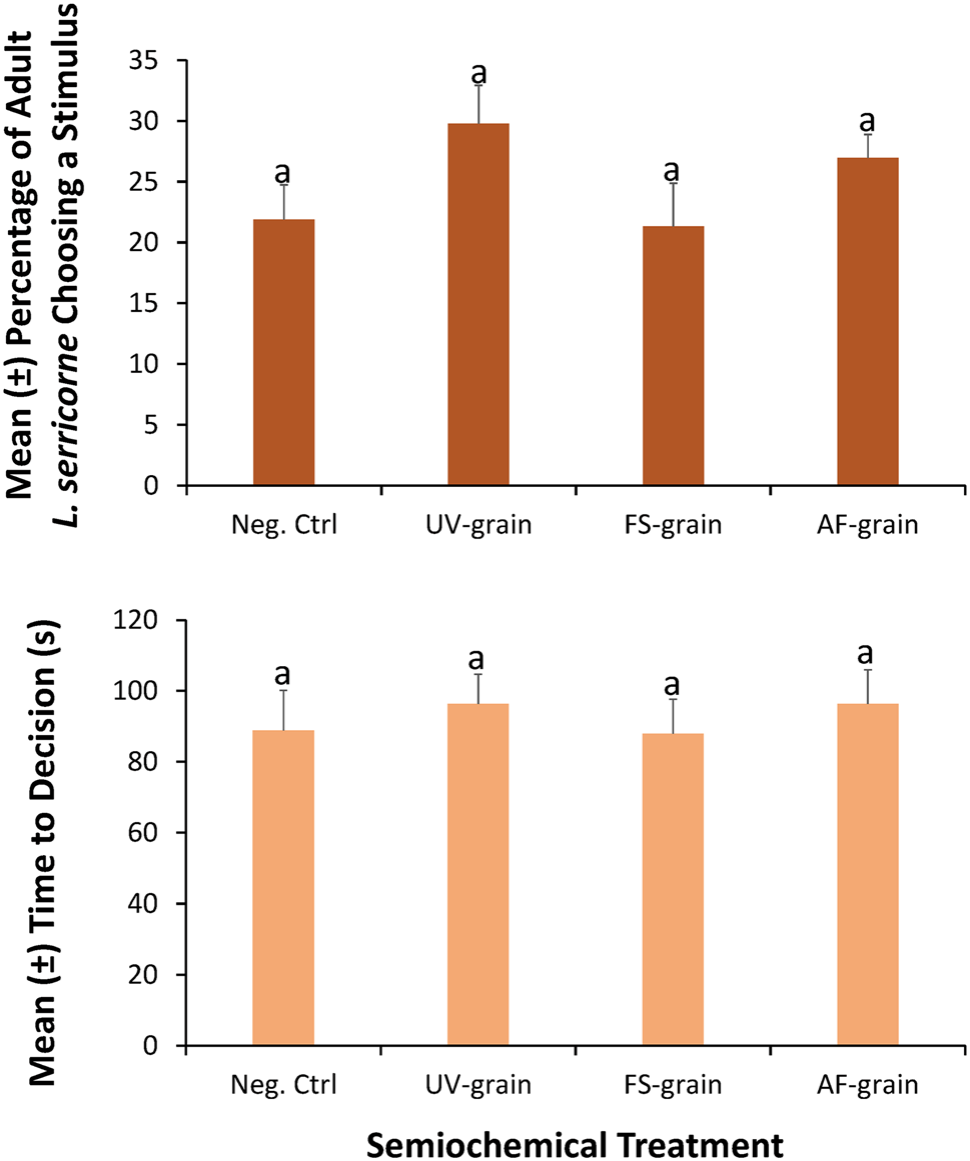
Preference shown in the four-way, still-air olfactometer using the mean percentage of *L. serricorne* adults choosing a stimulus (± SE) after given a 4-min opportunity (top panel) and their mean time to decision (± SE, s; bottom panel) based on the semiochemical treatment to which they were exposed in the laboratory at the USDA-ARS Center for Grain and Animal Health Research in Manhattan, KS. A total of n = 200 adults replicate were tested in this assay. Bars with shared letters are not significantly different from each other (Tukey HSD, α = 0.05). Neg. Ctrl, negative control; UV-grain, UV-sanitized grain; P. Ctrl, uninoculated grain; FS-grain, *F. verticillioides*-inoculated grain; AF-grain, *A. flavus*-inoculated grain.

The presence of the four stimuli did not significantly affect the percentage of adult *L. serricorne* choosing a treatment side in the four-way olfactometer (*χ*^*2*^ = 3.53; df = 3; *P* = 0.32; Fig. 1). Overall, *L. serricorne* chose UV-sanitized grain 30% of the time, while on average, they chose *Fusarium*-inoculated grain 21% of the time.

The semiochemical treatments in the four-way olfactometer had no significant effect on the time to decision by adult *L. serricorne* (ANOVA: *F* = 0.22; df = 3,174; *P* = 0.88). On average, decisions were made in 88–96 s by *L. serricorne* for the *Fusarium*-inoculated grain and *A. flavus*-inoculated grain, respectively.

### Movement assay overall measures

**Figure 2:**
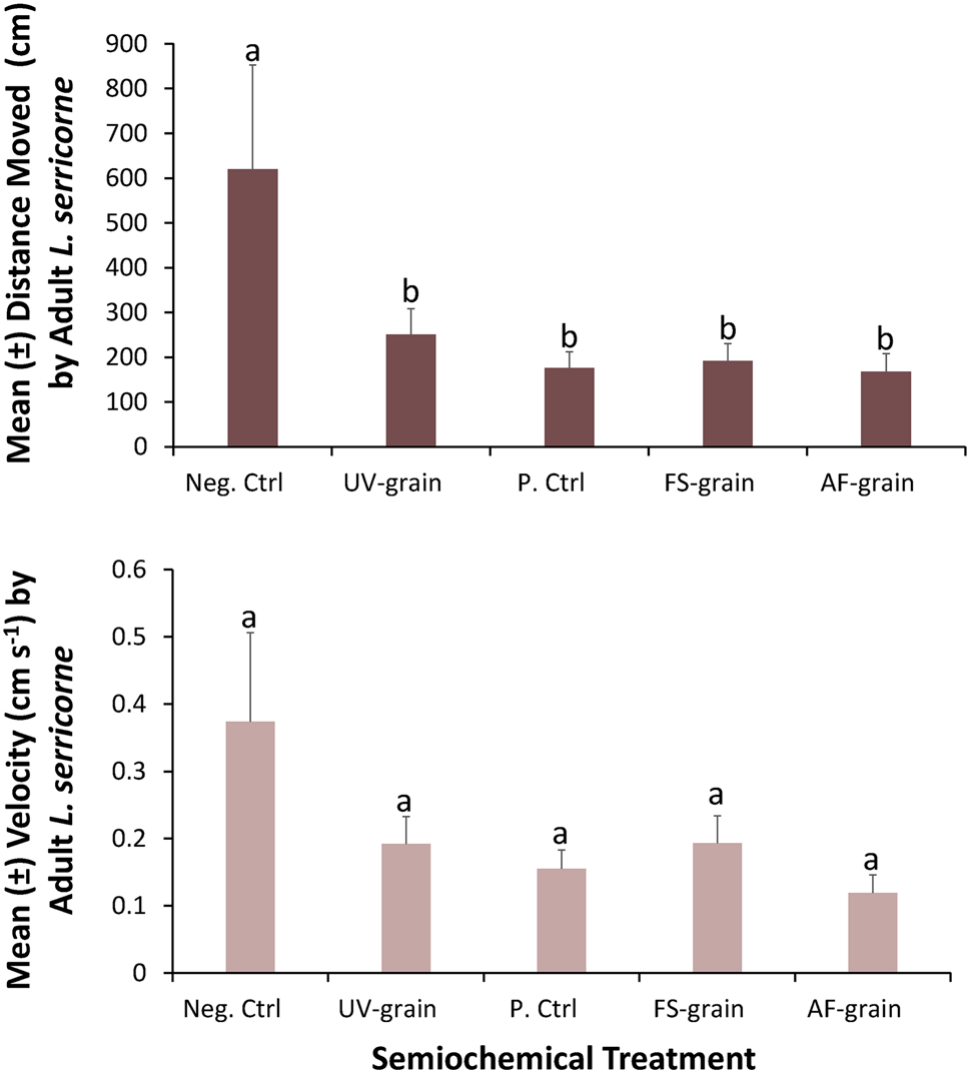
Metrics from the movement assay assessing the effects of MVOCs on mean distance moved (± SE, top panel) and the mean instantaneous velocity (± SE, bottom panel) by adult *L. serricorne* over a period of 30 min using a network camera coupled with Ethovision in the laboratory at the USDA-ARS Center for Grain and Animal Health Research in Manhattan, KS. There were a total of n = 20 replicates per treatment, translating to 50 h of data in this assay. Bars with shared letters are not significantly different from each other (Tukey HSD, α = 0.05). Neg. Ctrl, negative control; UV-grain, UV-sanitized grain; P. Ctrl, uninoculated grain; FS-grain, *F. verticillioides*-inoculated grain; AF-grain, *A. flavus*-inoculated grain.

The semiochemical treatments significantly affected the distance moved by adult *L. serricorne* over the 30-min observation periods (ANOVA: *F* = 541; df = 4, 86; *P* < 0.0001). Adult *L. serricorne* moved two to fourfold less when exposed to a grain kernel of one of the four semiochemical treatments compared to the negative control (Tukey HSD, Fig. 2). Adults moved numerically the least when exposed to *A. flavus*-inoculated grain kernels.

While velocity moved by *L. serricorne* showed the same numerical pattern in response to semiochemical treatments, it did not differ significantly (ANOVA: *F* = 1.28; df = 4, 86; *P* = 0.28). Overall, *L. serricorne* velocity was numerically decreased by two to threefold when exposed to a grain kernel of one of the four semiochemical treatments compared to the negative control (Fig. 2). The mean velocity ranged from 0.12 to 0.37 cm s^−1^ for the *A. flavus*-inoculated grain and negative control, respectively.

### Movement assay in kernel zones

**Figure 3:**
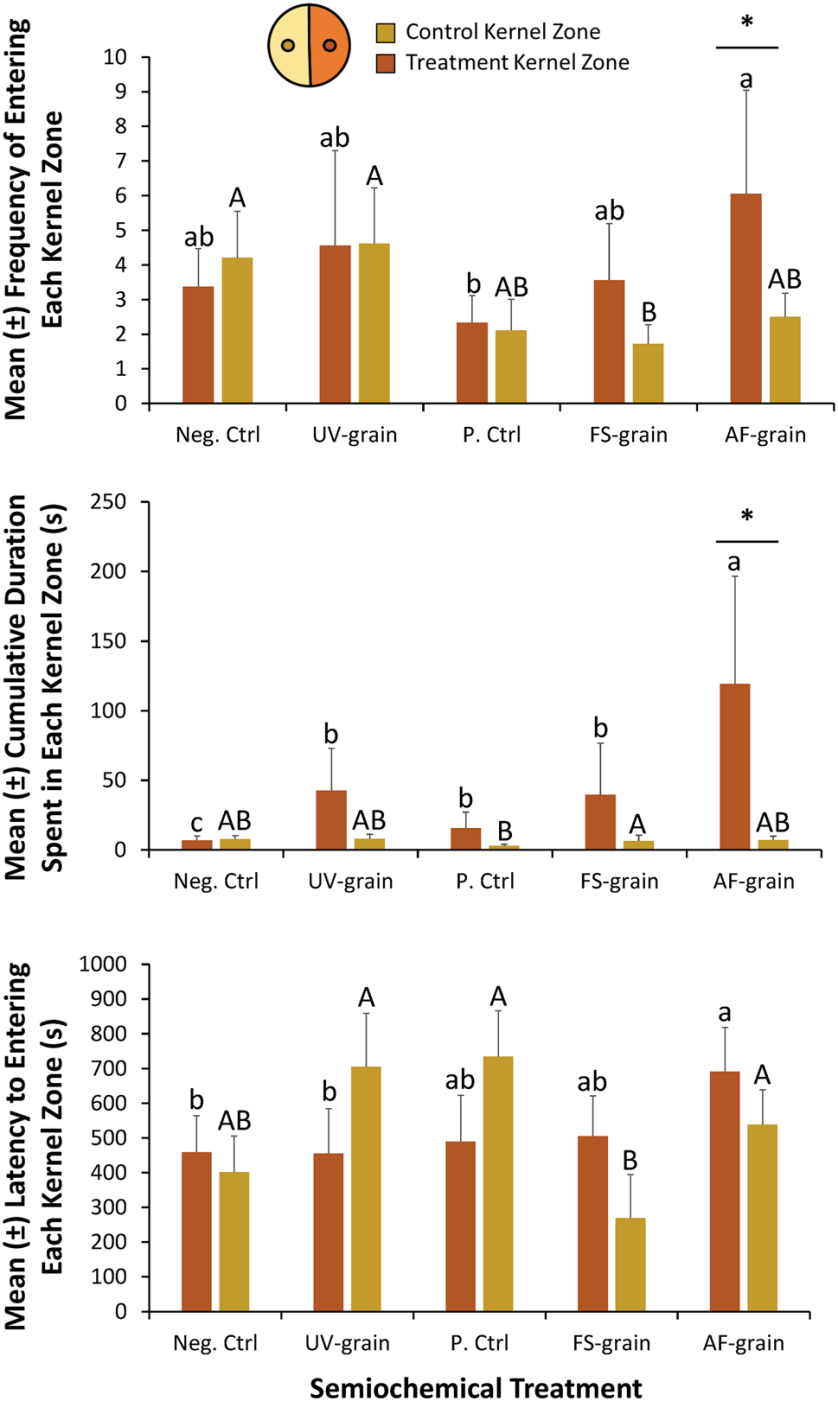
Treatment (dark orange bars) and control (dark yellow bars) kernel zone metrics from the movement assay, including determining how MVOCs affect the mean (± SE) frequency of entering each kernel zone (top panel), the cumulative duration spent in each kernel zone (middle panel), and the latency to finding and entering each kernel zone (bottom panel) by adult *L. serricorne* for a period of 30 min using video-tracking with a network camera coupled with Ethovision Software in the laboratory at the USDA-ARS Center for Grain and Animal Health Research in Manhattan, KS. The treatment kernel zone contains a single hard winter wheat kernel, while the control kernel zone does not contain any stimuli (neg. ctrl). There was a total of n = 20 replicate adults tested per treatment, translating to 50 h of data in this assay. Upper case letters represent multiple comparisons among levels of the control kernel zones only, while lower case letters represent multiple comparisons among levels of the treatment kernel zones only. Bars with shared letters are not significantly different from each other (Tukey HSD, α = 0.05). Asterisks (*) indicate post-hoc comparisons using paired t-tests (with Bonferroni correction), and only significant results have been displayed on applicable bars. Neg. Ctrl, negative control; UV-grain, UV-sanitized grain; P. Ctrl, uninoculated grain; FS-grain, *F. verticillioides*-inoculated grain; AF-grain, *A. flavus*-inoculated grain.

The semiochemical treatments significantly affected close-range foraging by altering the frequency by which an adult *L. serricorne* entered each kernel zone (MANOVA: *F* = 11.8; df = 4, 86; *P* < 0.0001; Fig. 3). The semiochemical treatments significantly affected the frequency by which an adult *L. serricorne* entered the control kernel zone, which lacked stimuli (ANOVA: *F* = 3.81; df = 4, 86; *P* < 0.01). Adult *L. serricorne* were two to threefold less likely to enter the control kernel zone in the *A. flavus*-inoculated grain and *Fusarium*-inoculated grain arenas compared to the negative control or UV-sanitized grain arenas (Fig. 3). Furthermore, the frequency of adult *L. serricorne* entering treatment kernel zones was significantly affected by the semiochemical treatment present (ANOVA: *F* = 6.01; df = 4, 86; *P* < 0.001). There were two to threefold more entries by adult *L. serricorne* to the treatment kernel zone containing the *A. flavus*-inoculated grain than to treatment kernel zones with uninoculated grain or no grain at all (Tukey HSD, Fig. 3). Importantly, *L. serricorne* entered treatment kernel zones with *A. flavus*-inoculated grain significantly more often than the associated control kernel zones (Post-hoc: *t* = 2.50; df = 19; *P* < 0.05; Fig. 3).

The semiochemical treatment significantly affected the cumulative duration spent in each kernel zone or arena half by adult *L. serricorne* (MANOVA: *F* = 51.9; df = 4,86; *P* < 0.0001). Particularly, the presence of the semiochemical treatments significantly affected the duration spent in both the control kernel zone (ANOVA: *F* = 3.19; df = 4,86; *P* < 0.05) and treatment kernel zone (ANOVA: *F* = 50.9; df = 4,86; *P* < 0.0001). The mean (± SE) cumulative duration spent in the control kernel zone was short and ranged from 3 (± 1.1) to 8 (± 3.0) s over the 30-min period. By contrast, *L. serricorne* spent 6–17-fold more time in the treatment kernel zone containing *Fusarium*-inoculated grain and *A. flavus*-inoculated grain, respectively, compared to the treatment kernel zone in the negative control. Adult *L. serricorne* spent the most time in the treatment kernel zone for UV-sanitized grain and *A. flavus*-inoculated grain, which was approximately 40 ± 28 and 119 ± 73 s, respectively. Importantly, *L. serricorne* spent significantly more time in the treatment kernel zone than the control kernel zone for *A. flavus*-inoculated grain (Post-hoc: *t* = 2.14; df = 19; *P* < 0.05).

The semiochemical treatments significantly affected the latency with which adult *L. serricorne* found each kernel zone or arena half (MANOVA: *F* = 44.7; df = 4, 86; *P* < 0.0001). For example, the semiochemical treatments significantly affected the latency to finding and entering the control kernel zone (ANOVA: *F* = 7.86; df = 4, 86; *P* < 0.0001) as well as the treatment kernel zone (ANOVA: *F* = 3.63; df = 4, 86; *P* < 0.01). Adult *L. serricorne* were two to threefold faster in finding *Fusarium*-inoculated grain control kernel zones than ones for UV-sanitized grain or uninoculated grain (Fig. 3). All semiochemical treatments in treatment kernel zones had a similar latency to being found and entered by *L. serricorne* except *A. flavus*-inoculated grain, which resulted in 1.5-fold higher latency than the other treatments.

### Movement assay in arena halves

**Figure 4:**
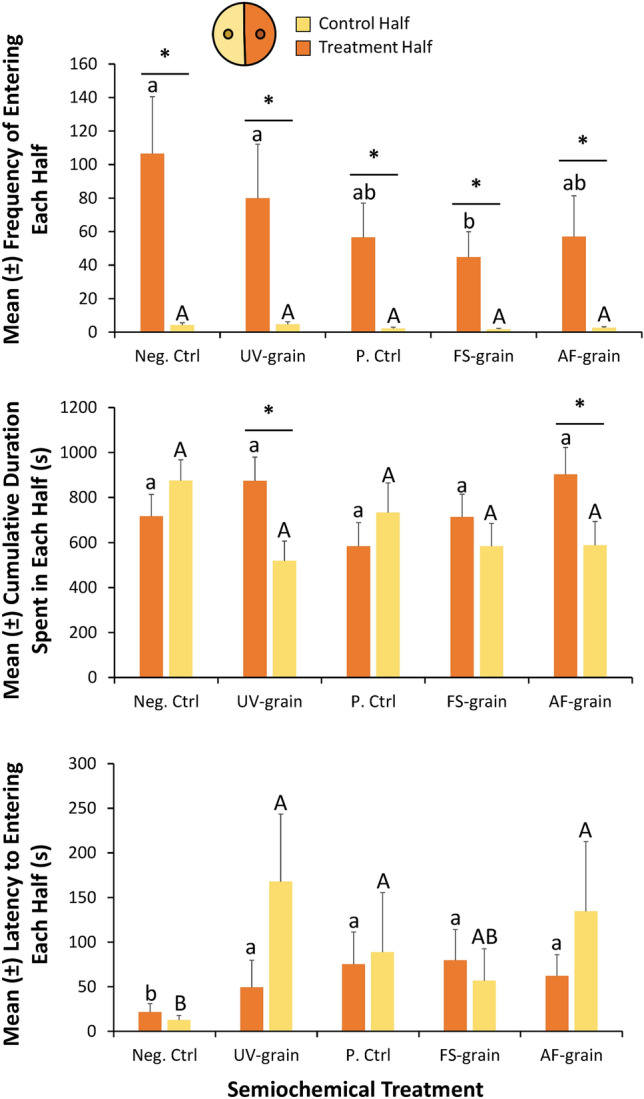
Treatment (orange bars) and control (yellow bars) zone metrics from the movement assay, including determining how MVOCs affect the mean (± SE) frequency of entering each zone (top panel), the cumulative duration spent in each zone (middle panel), and the latency to finding and entering each zone (bottom panel) by adult *L. serricorne* for a period of 30 min using video-tracking with a network camera coupled with Ethovision Software in the laboratory at the USDA-ARS Center for Grain and Animal Health Research in Manhattan, KS. The treatment zone contains a single hard winter wheat kernel, while the control zone does not contain any stimuli (neg. ctrl). There was a total of n = 20 replicate adults tested per treatment, translating to 50 h of data in this assay. Upper case letters represent multiple comparisons among levels of the control zones only, while lower case letters represent multiple comparisons among levels of the treatment zones only. Bars with shared letters are not significantly different from each other (Tukey HSD, α = 0.05). Asterisks (*) indicate post-hoc comparisons using paired t-tests (with Bonferroni correction), and only significant results have been displayed on applicable bars. Neg. Ctrl, negative control; UV-grain, UV-sanitized grain; P. Ctrl, uninoculated grain; FS-grain, *F. verticillioides*-inoculated grain; AF-grain, *A. flavus*-inoculated grain.

The frequency by which an adult *L. serricorne* entered the control half varied significantly among the semiochemical treatments (ANOVA: *F* = 5.21; df = 4, 86; *P* < 0.001). Adults entered control halves rarely, on average between 2 and 5 times over the 30 min period, corresponding to the *Fusarium*-inoculated grain and UV-sanitized grain treatment respectively (Fig. 4). Moreover, the frequency of entering the treatment half by an adult *L. serricorne* was significantly affected by the semiochemical treatment (ANOVA: *F* = 6.30; df = 4, 86; *P* < 0.001). There were each about twofold less frequent entries by adult *L. serricorne* to treatment halves with *A. flavus*-inoculated and *Fusarium*-inoculated grain compared to the negative control. Importantly, each semiochemical treatment in the treatment half resulted in significantly more entries than the corresponding control half (Post-hoc paired t-test, Bonferroni correction; Fig. 4).

By contrast, the semiochemical treatments did not significantly affect the cumulative duration spent in both control half (ANOVA: *F* = 1.12; df = 4,86; *P* = 0.35) nor in the treatment half (ANOVA: *F* = 0.81; df = 4,86; *P* = 0.52). Adult *L. serricorne* spent a range of 519–875 s in the control halves, on average, associated with the UV-sanitized and negative control treatment, respectively. Adults spent an average duration ranging from 584 to 902 s in the treatment halves, which corresponded to the uninoculated- and *A. flavus*-inoculated grain, respectively. Importantly, the cumulative duration spent in the *A. flavus*-inoculated treatment half was significantly more than the control half (Post-hoc: *t* = 2.01; df = 19; *P* < 0.05), as was the cumulative duration spent in the UV-sanitized treatment half compared to the control half (*t* = 2.56; df = 19; *P* < 0.01).

The semiochemical treatments significantly affected the latency to entering the control half (ANOVA: *F* = 28.1; df = 4,86; *P* < 0.0001) and treatment half (ANOVA: *F* = 13.7; df = 4,86; *P* < 0.0001) of the arenas. It took *L. serricorne* adults 4–13-fold longer to find control halves associated with UV-sanitized, uninoculated-, AF-inoculated grain, and *Fusarium*-inoculated grain semiochemical treatments compared to the negative control (Fig. 4). By contrast, *L. serricorne* were three to fourfold faster in finding the treatment halves associated with *Fusarium*-inoculated and *A. flavus*-inoculated grain compared to the negative control. Numerically, *L. serricorne* adults were over threefold faster in finding UV-sanitized treatment halves compared to control halves, while adults were twofold faster in finding *A. flavus*-inoculated treatment halves compared to control halves.

### Release-recapture assay

**Figure 5:**
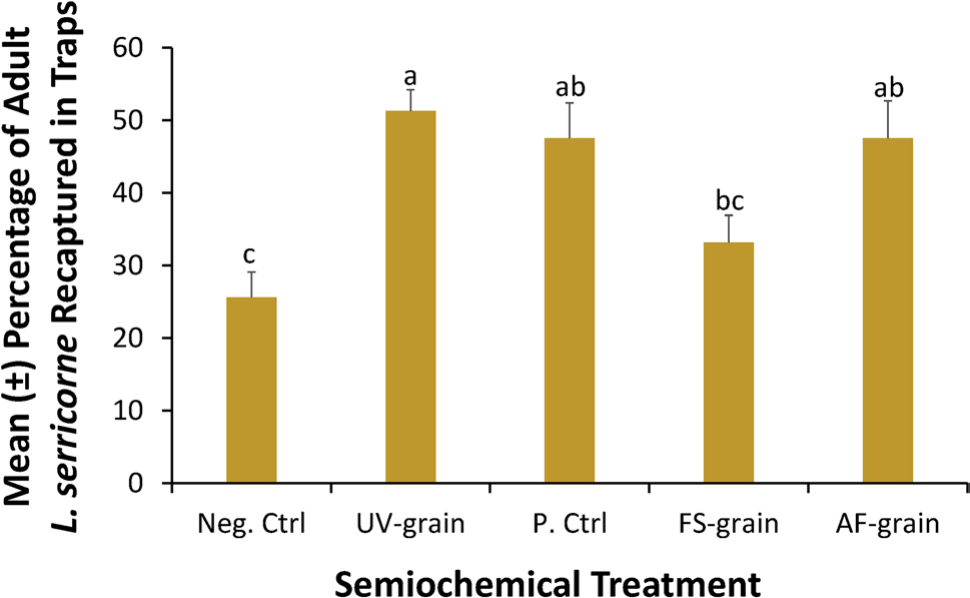
The mean percentage of adult *L. serricorne* recaptured in the release-recapture assay in response to MVOCs and grain used as lures in commercially-available monitoring pitfall traps, with a total of 20 adult, mixed-sex *L. serricorne* released in large plastic bins and placed in a large, walk-in environmental chamber under constant abiotic conditions and given 24 h to respond at the USDA-ARS Center for Grain and Animal Health Research in Manhattan, KS for a total of n = 8 replicate releases per treatment. Bars with shared letters are not significantly different from each other (Tukey HSD, α = 0.05).

In total, the percent of adults recaptured out of 800 was 41%, ranging between 26 and 51%. The attractant in the trap significantly affected recapture of *L. serricorne* in commercially-available pitfall traps (ANOVA: *F* = 7.11; df = 4,35; *P* < 0.001; Fig. 5). In fact, the greatest recapture was in traps baited with UV-sanitized, uninoculated-, and *A. flavus*-inoculated grain, which each captured approximately twofold more *L. serricorne* than the negative control. Traps baited with *Fusarium*-inoculated grain captured an intermediate percentage of *L. serricorne*.

### Headspace volatiles collected from inoculated grain

**Figure 6:**
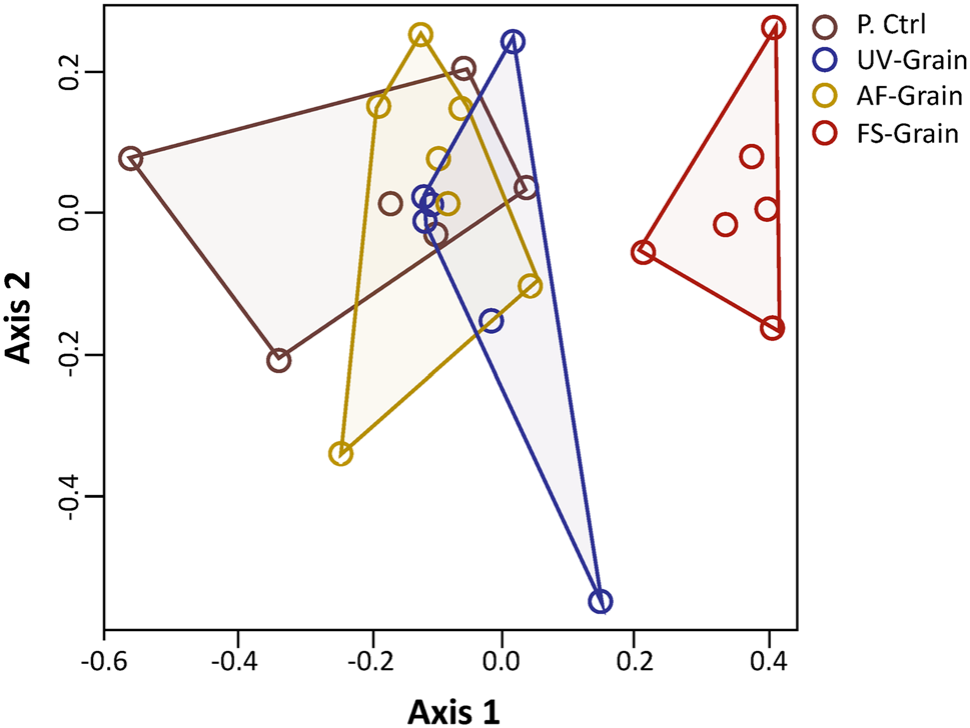
Non-metric multi-dimensional scaling ordination plot based on pairwise Bray–Curtis similarity index calculated between each combination of volatile emissions (ng h^−1^ g^−1^ grain) by samples after headspace collection for 3 h from 20 g of grain that was UV sanitized for 10 min (blue circles), inoculated with *A. flavus* (yellow circles), inoculated with *Fusarium* spp (red circles), or uninoculated (brown circles). There were n = 7 replicates per semiochemical treatment. Polygons represent convex hulls drawn between samples and are color coded similarly to treatments. There were a total of n = 1000 permutations, and stress was < 0.10, enabling adequate interpretation. UV-grain, UV-sanitized grain; P. Ctrl, uninoculated grain; FS-grain, *F. verticillioides*-inoculated grain; AF-grain, *A. flavus*-inoculated grain.

**Figure 7:**
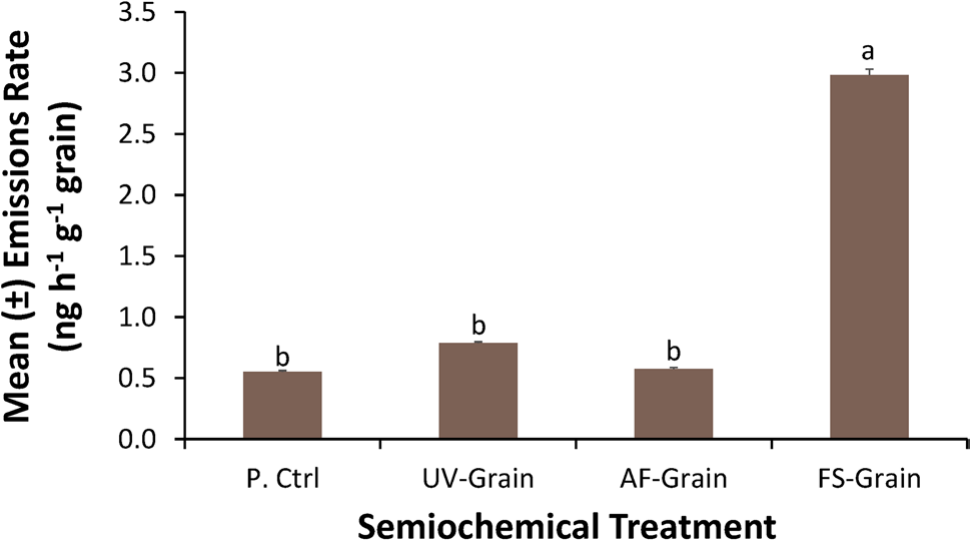
Mean emissions rate from the semiochemical treatments (in ng h^−1^ g^−1^ grain) during headspace collection in 500-mL capacity containers after adsorption to Porapaq-Q volatile collection trap, then elution with 150 µL dichloromethane. Bars with shared letters are not significantly different from each other (Tukey HSD, α = 0.05). UV-grain, UV-sanitized grain; P. Ctrl, uninoculated grain; FS-grain, *F. verticillioides*-inoculated grain; AF-grain, *A. flavus*-inoculated grain.

In total, 44 compounds were tentatively identified from the semiochemical treatments (Table 1). The uninoculated grain had the fewest number of compounds (e.g. just 15), while the UV-sanitized grain, *A. flavus*-inoculated grain, and *Fusarium*-inoculated grain each had 23, 26, and 25 compounds in its headspace, respectively (Table 1). There was some overlap, but significant differentiation among treatments in volatile composition (ANOSIM: *R* = 0.305; Perm = 1000; *P* < 0.0001; Fig. 6). Many of the compounds occurring in the *A. flavus*- and *Fusarium*-inoculated grain were unique compared to the uninoculated- or UV-sanitized grain (Table 1), including 2-(1-propenyl)-6-methylphenol-butanoic acid, cyclopropanecarboxylic acid, and octane for *A. flavus*-inoculated grain and 2-epi-α-cadrene, (1*R*,4*S*,5*S*)-1,8-dimethyl-4-(prop-1-en-2-yl)spiro[4.5]dec-7-ene, and 4-ethyl-1,2-dimethoxy-benzene for *Fusarium*-inoculated grain. Finally, there was significantly higher total emissions by *Fusarium*-inoculated grain compared to the uninoculated grain (*t* = 47.7; df = 86; *P* < 0.0001), UV-sanitized grain (*t* = 42.9; df = 86; *P* < 0.0001), and *A. flavus*-inoculated grain (*t* = 47.1; df = 86; *P* < 0.0001), with 5-, 4-, and 5-fold higher emissions, respectively (Fig. 7).

**Table 1:** Summary of headspace volatiles emitted (in ng h^−1^ g^−1^ grain) from grain that was uninoculated (P. ctrl), surface sanitized with UV-radiation for 10-min (UV-grain), or inoculated with *A. flavus* or *Fusarium* spp. for periods of 3 h using a push headspace collection system.

Compound	RT	P. ctrl		UV-grain		*A. flavus*-grain		*Fusarium*-grain	
		Mean ± SE	% of Total	Mean ± SE	% of Total	Mean ± SE	% of Total	Mean ± SE	% of Total
4-Ethylbenzamide	4.26	0.00 ± 0.00	0	0.00 ± 0.00	0	0.00 ± 0.00	0	0.17 ± 0.11	6
cis-1-Butyl-2-methylcyclopropane	4.38	0.10 ± 0.06	18	0.03 ± 0.01	4	0.04 ± 0.02	6	0.01 ± 0.02	0
3-Octene, (E)^a^	4.38	0.00 ± 0.00	0	0.02 ± 0.01	3	0.02 ± 0.02	3	0.00 ± 0.00	0
4-Heptanone^a^	4.45	0.01 ± 0.01	2	0.02 ± 0.01	2	0.01 ± 0.01	2	0.03 ± 0.04	1
Octane^a^	4.46	0.00 ± 0.00	0	0.00 ± 0.00	0	0.02 ± 0.03	3	0.05 ± 0.07	2
2,4-Dimethyl-1-heptene	4.91	0.01 ± 0.01	3	0.05 ± 0.02	7	0.02 ± 0.02	4	0.03 ± 0.03	1
2-Pentanone, 4-hydroxy-4-methyl	4.92	0.03 ± 0.02	6	0.05 ± 0.04	6	0.03 ± 0.04	5	0.00 ± 0.00	0
3-Hexanone, 2-methyl^a^	5.26	0.00 ± 0.00	0	0.00 ± 0.00	0	0.00 ± 0.00	0	0.03 ± 0.04	1
Pyrazole-1-methanol	5.63	0.00 ± 0.00	0	0.03 ± 0.02	3	0.01 ± 0.01	1	0.00 ± 0.00	0
Mesitylene^a^	6.62	0.00 ± 0.00	0	0.00 ± 0.00	0	0.00 ± 0.00	0	0.06 ± 0.07	2
Pentane, 2,2,3,3-tetramethyl	7.09	0.03 ± 0.01	6	0.06 ± 0.02	8	0.02 ± 0.02	4	0.04 ± 0.03	1
Octane, 3,4,5,6-tetramethyl	7.15	0.04 ± 0.01	7	0.03 ± 0.01	4	0.05 ± 0.03	9	0.13 ± 0.13	4
Hexanoic acid, 2-ethyl-, methyl ester	7.57	0.00 ± 0.00	0	0.00 ± 0.00	0	0.00 ± 0.00	0	0.12 ± 0.06	4
Ether, hexyl pentyl	7.89	0.00 ± 0.00	0	0.01 ± 0.01	1	0.01 ± 0.01	1	0.00 ± 0.00	0
3-Hydroxy-3-methylvaleric acid	7.89	0.01 ± 0.01	2	0.00 ± 0.00	0	0.00 ± 0.00	0	0.06 ± 0.05	2
Benzene, 1,4-diethyl	7.89	0.00 ± 0.00	0	0.01 ± 0.01	2	0.00 ± 0.00	0	0.00 ± 0.00	0
Phenol, 3,4,5-trimethyl	8.13	0.00 ± 0.00	0	0.01 ± 0.01	2	0.01 ± 0.01	1	0.15 ± 0.05	5
Ethanol, 2-(octyloxy)	8.14	0.01 ± 0.01	1	0.00 ± 0.00	0	0.01 ± 0.01	1	0.00 ± 0.00	0
1-Undecene, 7-methyl^a^	8.20	0.00 ± 0.00	0	0.01 ± 0.01	1	0.02 ± 0.01	3	0.00 ± 0.00	0
1-Dodecanol^a^	8.21	0.00 ± 0.00	0	0.01 ± 0.01	1	0.01 ± 0.01	2	0.00 ± 0.00	0
Undecane^a^	8.41	0.04 ± 0.03	7	0.03 ± 0.03	4	0.02 ± 0.03	3	0.01 ± 0.01	0
Nonanal^a^	8.51	0.06 ± 0.04	11	0.06 ± 0.05	7	0.03 ± 0.03	5	0.00 ± 0.00	0
Cyclopropanecarboxylic acid	8.52	0.00 ± 0.00	0	0.00 ± 0.00	0	0.02 ± 0.03	3	0.25 ± 0.26	8
5-Methylthiopyridin-2-ol	9.17	0.04 ± 0.03	8	0.03 ± 0.02	4	0.00 ± 0.00	0	0.03 ± 0.04	1
1-Hexanol, 5-methyl-2-(1-methylethyl)	10.54	0.00 ± 0.00	0	0.00 ± 0.00	0	0.00 ± 0.01	1	0.00 ± 0.00	0
4-Phenyl-2-butanol^a^	10.65	0.00 ± 0.00	0	0.03 ± 0.03	3	0.00 ± 0.00	0	0.00 ± 0.00	0
Benzene, 1,3-bis(1,1-dimethylethyl)-	10.78	0.09 ± 0.03	16	0.16 ± 0.05	20	0.13 ± 0.04	22	0.11 ± 0.06	4
Ethanone, 1-(4-ethylphenyl)-	10.96	0.00 ± 0.00	0	0.00 ± 0.00	0	0.00 ± 0.00	0	0.00 ± 0.00	0
Butanoic acid, 2-(1-Propenyl)-6-methylphenol-	11.04	0.00 ± 0.00	0	0.00 ± 0.00	0	0.02 ± 0.02	4	0.00 ± 0.00	0
m-Ethylacetophenone-^a^	11.25	0.00 ± 0.00	0	0.08 ± 0.07	10	0.00 ± 0.00	0	0.00 ± 0.00	0
2,3,6-Trimethylhept-3-en-1-ol -	11.54	0.00 ± 0.00	0	0.00 ± 0.00	0	0.01 ± 0.01	2	0.00 ± 0.00	0
Ketone, methyl 2,2,3-trimethylcyclopentyl	11.66	0.00 ± 0.00	0	0.00 ± 0.00	1	0.01 ± 0.01	1	0.00 ± 0.00	0
Dodecane, 4-methyl-	11.67	0.02 ± 0.01	3	0.00 ± 0.00	0	0.01 ± 0.01	2	0.00 ± 0.00	0
Benzene, 4-ethyl-1,2-dimethoxy-	11.68	0.00 ± 0.00	0	0.00 ± 0.00	0	0.00 ± 0.00	0	0.49 ± 0.31	16
.alpha.-Cubebene	13.22	0.00 ± 0.00	0	0.00 ± 0.00	0	0.00 ± 0.00	0	0.08 ± 0.05	3
Germacrene D	13.37	0.00 ± 0.00	0	0.00 ± 0.00	0	0.00 ± 0.00	0	0.16 ± 0.08	5
(1R,4S,5S)-1,8-Dimethyl-4-(prop-1-en-2-yl)spiro[4.5]dec-7-ene	13.82	0.00 ± 0.00	0	0.00 ± 0.00	0	0.00 ± 0.00	0	0.40 ± 0.15	13
Di-epi-.alpha.-cedrene	15.89	0.00 ± 0.00	0	0.00 ± 0.00	0	0.00 ± 0.00	0	0.35 ± 0.17	12
Benzimidazole, 2-ethyl-1-propyl-	18.42	0.00 ± 0.00	0	0.00 ± 0.00	0	0.00 ± 0.00	0	0.03 ± 0.02	1
Hexanedioic acid, bis(2-ethylhexyl) ester	23.11	0.04 ± 0.04	7	0.03 ± 0.03	4	0.03 ± 0.05	5	0.11 ± 0.12	4
Total emissions (ng per h per g grain)		0.6 ± 0.01	100	0.8 ± 0.01	100	0.58 ± 0.01	100	3.0 ± 0.05	100

There were n = 7 replicates per treatment, and mean emission rates per compound are portrayed.

^a^Compound identity confirmed by co-injecting technical standard onto same column.

## Discussion

Overall, this is the first study to examine in an in-depth manner how specific fungal species affect the behavior of adult *L. serricorne*, particularly how MVOCs from fungi may be used as foraging cues to manipulate insect attraction, mobility, and preference in the post-harvest environment. We included multiple behavioral assays (e.g., four-way olfactometer for preference, video-tracking to evaluate close-range foraging decisions, and a release-recapture assay to determine long-distance attraction) all to ultimately characterize how *L. serricorne* responds to MVOCs and interacts with microbes. Here, we show that inoculating grain with known plant pathogens has direct implications on the MVOCs produced, and in turn, this affects the behavior of an external-infesting stored product pest, *L. serricorne*. We found that this is true at both close-range and at distance. This may be a result of the fact that *L. serricorne* evolved from a lineage of insects that likely infested animal caches^4,36^, which are hypothesized to preferentially be attracted to MVOCs as a reliable host cue. MVOCs may be reliable cues in this situation because animals often forget or do not use many of their caches^41^, leaving them to mold in temperate environments. It may be possible *L. serrcorne* responds specifically to *A. flavus* due to its prevalent frequency in flour^25^, and its propensity especially for xeric conditions^28^, which is a propensity shared by *L. serricorne*^11^. By contrast, *F. verticillioides* prefers more moist or humid environments. While there is abundant data that there are close symbioses between *L. serricorne* and *Symbiotaphrina*, there is no support for this for *A. flavus* and *F. verticillioides*, which we isolated from the beetle cuticle. It is more likely, these fungi were already present on the grain, and *L. serricorne* acted as a physical vector, though certainly the microbial community has been known to create microclimates in grain and have an effect on the insect community under some conditions (Ponce and Morrison, unpublished data). Together, this presents a convincing case for the importance of insect-microbe interactions on the foraging ecology of *L. serricorne*. Below, we go over each of the main results in turn.

We found close-range foraging by *L. serricorne* is affected by grain and MVOCs. Altogether, the volatile emissions from inoculated grain by the fungal morphotype significantly affects the foraging decisions by *L. serricorne* both when in close-proximity and at a distance to the inoculated grain as compared to uninoculated and sanitized grain. In fact, this supports prior work showing that there are a variety of ecological and behavioral responses by insects to volatile emissions from microbes^7^. At close-range, *L. serricorne* used volatiles from *Aspergillus flavus*-inoculated grain for foraging decisions, and this was reflected in some differentiation in the volatile composition from the *A. flavus* treatment compared to the other treatments. Similarly, another stored product pest, *S. oryzae*, oriented to MVOCs from *A. flavus*-inoculated grain at close-ranges, but preferred to use only grain volatiles at a distance^10^. Interestingly, our results also showed that grain without microbes (i.e. UV-sanitized grain) was also an attractive source for *L. serricorne* at close-range and a distance. In particular, our work showed that the presence of UV-sanitized and *A. flavus*-inoculated grain increased the cumulative duration spent in and the frequency of entering each of those kernel zones. Once *L. serricorne* entered the UV-sanitized or microbial-inoculated kernel zones, their movement was arrested. In the experiment, mixed-sex adults were used because of difficulty distinguishing sexes as adults. However, males and females may be under different selection pressures. For example, males may be more motivated to disperse, and as such may seek advantages conferred from microbial partners to use intact grain that they may not otherwise have access to (mentioned as a hypothesis in Ponce et al.^4^). By contrast, females may be under selective pressure to find optimal oviposition locations that would be free from microbial contamination for progeny. Follow-up work could find support for this hypothesis by sexing *L. serricorne* when they are pupae, then repeating the experiment to determine if there are differences in response to these two stimuli that break down along sex-based lines. In terms of food kairomones that affect foraging, volatiles from *Capsicum* products were found to be significantly more attractive to *L. serricorne* than products without *Capsicum* (e.g., cracked wheat, corn, tobacco, mineral oil, grape seed oil, rolled oats, coriander seeds, and sesame oil)^42^. In prior work, the fungal volatile, 1-octen-3-ol was a strong attractant for the stored product beetle, *Ahaversus advena* (Waltl) (Coleoptera: Silvanidae)^43^ supporting the contention that stored product insects use MVOCs in foraging decisions. Overall, our work confirms that *L. serricorne response* to MVOCs is microbe species-dependent, and volatiles from *A. flavus*-inoculated grain elicit a behavioral response at close- and long-range by the insect.

Here, we found that adult *L. serricorne* moved two to fourfold less and their velocity was numerically decreased by 2–threefold when exposed to a grain kernel of one of the four semiochemical treatments compared to the negative control. This suggests that one of the key processes happening at close range in response to the grain is arrestment, demonstrated by slower and less movement of *L. serricorne* in response to the semiochemicals. Prior work has documented the sublethal effects of insecticide, dispersal, and foraging using distance moved and velocity as surrogates of movement^44^ that can be mediated by good and poor sanitation^45^. However, prior work has rarely evaluated response to MVOCs using velocity or distance moved. Nevertheless, Lizarraga^46^ found a numeric decrease in the distance moved and velocity when *S. oryzae* were exposed to the uninoculated grain, but this was not significantly different from the negative control or any other treatment. McFarlane^47^ used Ethovision and found an important stored product pest, the red flour beetle, *T. castaneum* most often spent time in a wind tunnel adjacent to an empty arena instead of one with a stimulus containing the fungus, *Aspergillus tubingensis*. Most other work with Ethovision has been on the sublethal effects of insecticides^48–52^ demonstrating an overlooked tool in understanding the effect of MVOCs on the behavioral and chemical ecology of stored product pests.

While prior studies have directly assessed response to pure fungal volatiles bought from a supplier or synthesized^43,53–55^, our study links inoculation of grain by specific microbes with changes in MVOC headspace profiles and determined the implications for *L. serricorne* behavior. We found a total of 44 tentatively identified, but distinct principal compounds in the headspace emissions from clean and fungal-inoculated grain. There were unique cues in the fungal-inoculated grain, but fungal identity, and by consequence, compound identity appears to matter for behavioral response by *L. serricorne*. *Fusarium verticillioides*-inoculated grain was significantly different than the other treatments, yet it did not elicit the same change in behavior as the *A. flavus*-inoculated grain, which showed more overlap with the other treatments. Thus, minor compounds uniquely emitted by or emitted in unique ratios by *A. flavus*, but not present in the other treatments (Table 1), may be important for the behavioral response of *L. serricorne*. For example, both 5-methyl-2-(1-methylethyl)-1-hexanol and 2-(1-propenyl)-6-methylphenol-butanoic acid were only present in *A. flavus*-inoculated grain and not in any other treatment. Interestingly, Ponce et al.^4^ found that 3-octanone, 1-octen-3-ol, octane, 3-methyl-1-butanol and many others were the most commonly tested MVOCs for behavioral effects against stored-product arthropods, and we found a subset of these in the headspace emissions here. Similarly, Sinha^56^ found 3-methyl-1-butanol, 1-octen-3-ol, and 3-octanone were the most common compounds isolated in experimental grain bins, which supports some of the headspace volatiles emitted from grain in our experiment. In addition, many of the compounds occurring in the *A. flavus*- and *Fusarium*-inoculated grain were unique compared to the uninoculated- or UV-sanitized grain (Table 1), including 2-(1-propenyl)-6-methylphenol-butanoic acid, cyclopropanecarboxylic acid, and octane for *A. flavus*-inoculated grain and 2-epi-α-cadrene, a long-chain alkene, and 4-ethyl-1,2-dimethoxy-benzene for *Fusarium*-inoculated grain. Overall, the total emission rate for volatiles produced by *Fusarium*-inoculated grain is three to fivefold several magnitudes higher, than the uninoculated grain. While overall volatile emissions among fungal-inoculated grain were similar, the identity of the volatiles varied. This may lead to the differences observed insect behavior. In the future, complex blends of MVOCs emitted by common fungi should be evaluated against stored product insects using gas chromatography coupled with electroantennography (GC-EAD) in order to determine which volatiles are perceived by individuals. Further investigation of the MVOCs on foraging behavior of stored product insects may lead to promising candidate compounds that are behaviorally-active to manipulate pest populations in management tactics at food facilities^36,57^.

No preference was found by *L. serricorne* among the semiochemical stimuli including UV-sanitized grain, uninoculated grain, *F. verticillioides*-inoculated grain, *A. flavus*-inoculated grain and the negative control using the four-way olfactometer. This could be because there may have been a thigmotactic reaction to the glass substrate by *L. serricorne*. Other stored product species have been documented to exhibit thigmotactic responses to netting, for example^58^, though this has not been documented for *L. serricorne*. Additionally, it is possible that while some of the treatments have attractant or arresting properties as individual bouquets, complex blends admixing in the headspace of the release chamber may result in no preference among each other. Prior work has confirmed that volatiles are able to quickly and adequately diffuse by Brownian motion over the allotted trial period in the assay^10^. Thus, performance of this assay in the biosafety cabinet did not prevent volatiles from reaching test subjects, and was a valid test of the odor treatments. Prior work found that the invasive stored product dermestid *Trogoderma granarium* Everts (Coleoptera: Dermestidae) showed attraction to pheromonal and kairomonal stimuli that were not perfectly congruous with the results from work in related preference assays for the same stimuli^59^. Gerken et al.^60^ found that even with the same species (e.g., *T. castaneum*), one could obtain different results using multiple behavioral assays, and suggested that one must carefully select the assays and consider the actual variables being tested. However, the lack of a positive response in only one out of the multiple assays to headspace by *A. flavus* in this study suggests that there is a relatively robust response to the mVOCs from this microbial species by *L. serricorne*.

In the future, research should be prioritized to (1) address which compounds identified in Table 1 are actually detected by *L. serricorne* through GC-EAD, (2) follow-up on GC-EAD results with behavioral tests to determine whether the compounds are attractive, repellent, or neutral, and (3) formulate new lures with the most attractive compounds to improve monitoring and management programs*.* This will help narrow the behaviorally-active components of the headspace blends to those that are being detected by the insect. Overall, this study has enhanced our understanding of how microbially-produced volatiles organic compounds from postharvest fungal grain pathogens affect the behavior of *L. serricorne*. Increasingly, it is apparently essential to manage microbes and insects simultaneously in the postharvest agricultural supply to ensure that our planet can feed its growing population.

## Materials and methods

### Source insects

Adult cigarette beetles, *Lasioderma serricorne* (F.) (Coleoptera: Ptinidae)*,* were used from insect colonies that were originally collected in 2012 from a rice mill in Arkansas and continuously maintained on a diet of 95% bleached wheat flour and 5% brewer’s yeast with oats sprinkled on top and a moistened, crumpled towel added. Colonies were kept at the United States Department of Agriculture Agricultural Research Service’s (USDA-ARS) Center for Grain and Animal Health Research facility in Manhattan, KS. Insects were routinely subcultured by sieving (No. 30 sieve, 594 × 594 µm mesh, W.S. Tyler Co., Cleveland, OH, USA) 150 mixed-sex *L. serricorne* adults from diet mixtures, transferring them into new mason jars (950-ml capacity) filled two-thirds of the way to the top with diet, and then placed inside environmental chambers under constant conditions at 27.5 °C, 65% RH, and 14:10 L:D photoperiod. Individuals used in bioassays described below were never tested more than once.

### Fungal morphotype culturing

To initially isolate the two fungal morphotypes, *L. serricorne* were allowed to disperse on agar. A total of 32 g of potato dextrose agar (Merck, Darmstadt, Germany) was mixed with 900 ml of deionized water in a 1000-mL glass media bottle with a magnetic stirring rod placed inside the bottle. The agar solution was autoclaved (533LS, Getinge, Rochester, NY, USA) for 30 min and then stirred on a hot plate to cool down for 20 min. Before pouring the potato agar solution into petri dishes (100 × 15), the biosafety cabinet (75 × 73 × 95 cm L:H:W, #302381101, Labconco, Kansas City, MO, USA) was sanitized with 70% ethanol and exposed to UV light for 10 min. A total of 36 petri dishes with potato dextrose agar solution were left in the biosafety cabinet to solidify overnight.

**Figure 8:**
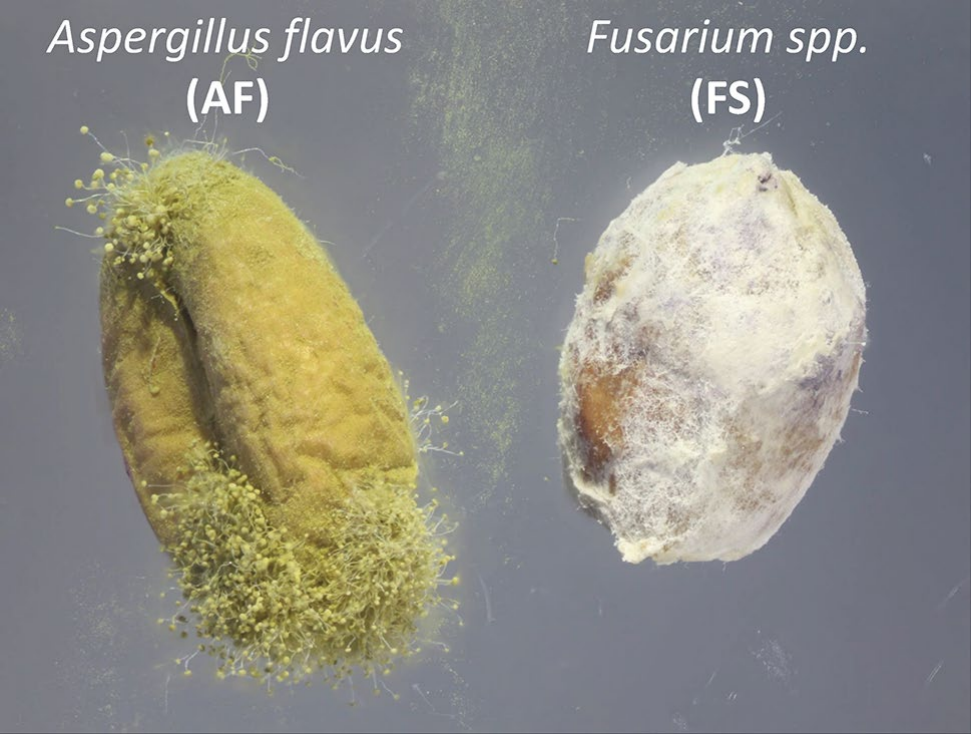
Habitus images of morphotype 1 (left, *A. flavus*-inoculated grain) and morphotype 2 (right, *F. verticillioides*-inoculated grain) at ×10 magnification (SMZ-18, Nikon Inc., Tokyo, Japan) on grain after inoculation procedure.

A single *L. serricorne* was introduced into to a single PDA dish and microbial growth was visualized after 3 and 5 d. Each PDA plate was sealed with parafilm, and stored in an environmental chamber under constant conditions of 30 °C, 60% RH, and 14:10 L:D photoperiod. Transfer of *L. serricorne* from containers to agar was performed inside the biosafety cabinet to prevent contamination. Pictures of the agar dishes and corresponding microbial growth were acquired using a DSLR camera (EOS 7D Mark II, Canon, Tokyo, Japan) mounted to 3D imaging StackShot (CogniSys, Inc., Traverse City, MI, USA) equipped with a dual flash (MT-26EX-RT, Canon, Tokyo, Japan). Light was diffused using a partially cut frosted plastic jar (15.2 × 7.6 cm D:H) making a total of 12 replicates. From the fungal isolation, two fungal morphotypes were selected by close examination of microbial characteristics such as spore shape, size, and color. Morphologically, *A. flavus* often appears yellowish or green in pigment, while *F. graminearum* often appears white (Fig. 8). This was used to generate the two primary morphotypes for the rest of the assays in the experiments below, and to generate sequences for the morphotypes.

### Treatments

To study how MVOCs affected *L. serricorne* behavior, the two morphotypes identified above were introduced to grain and compared to uninoculated and UV-sanitized grain in various behavioral assays. The uninoculated grain treatment (e.g., P. ctrl hereafter) consisted of 10 g of hard winter wheat at a 10.6% moisture content taken directly from cold storage (Table 2). UV-sanitized grain was as above but treated with ultraviolet radiation for 10 min in the biosafety cabinet (75 × 73 × 95 cm L:H:W, #302381101, Labconco, Kansas City, MO, USA) to surface sterilize. Grain inoculated by specific fungal species included *Fusarium-*inoculated grain (e.g. *F. verticillioides*, determined by sequencing, see below: FS, hereafter) and *A. flavus*-inoculated grain (AF, hereafter) (Fig. 8). In order to develop uniform, inoculated cultures on grain, our process was modelled on mushroom farming techniques with boiling^61^. To prepare the *Fusarium*-inoculated grain, 600 g of grain was transferred to a stainless-steel pot filled with water and placed on a hot plate at 100 °C. Once boiling for 15 min, the water was drained and the grain was evenly spread out on sterile wipes (38.1 × 42.5 cm, 3 ply, Tech wipes, Skilcraft, NIB, Alexandria, VA) and dried inside a laminar flow hood (ca. 3 h). Afterwards, grain was evenly divided (~ 300 g) and placed in 2 separate autoclaved mason jars (950-mL capacity). A single hole was pierced through each lid and lined with a cotton ball. The jars were then sealed with aluminum foil and were autoclaved (533LS, Getinge, Rochester, NY, USA) for 30 min. To inoculate with *Fusarium* spp. or *A. flavus*, a 3-inch agar plug of fungi was placed into each jar containing the grain. The plug had been colonized and growing without contamination in a petri dish for 7–10 days in an environmental chamber set to 30 °C, 60% RH, and 14:10 L:D photoperiod (Supplementary Fig. 2). Jars with freshly prepared *Fusarium*-inoculated grain sat at room temperature for roughly 10–15 days or until the fungi evenly covered as much of the grain as possible. Grain was never used more than once for each replicate of every trial in each assay experiment to prevent cross contamination.

**Table 2:** List of semiochemical treatments and the amounts used in each assay during the study.

Abbrev.	Treatments	Amount in assay			
		4-way Olfact	Ethovision	Headspace	Release-recapture
Neg. Ctrl	Negative control	0	0	0	0
P. Ctrl	Positive control (uninoculated)	–	1 kernel	20 g	10 g
AF-grain	*Aspergillus flavus*-inoculated	10 g	1 kernel	20 g	10 g
FS-grain	Fusarium *verticillioides*-inoculated	10 g	1 kernel	20 g	10 g
UV-grain	UV-surface sanitized for 10 min	10 g	1 kernel	20 g	10 g

### Microbial morphotype sequencing

To identify microbes associated with the cuticle of *L. serricorne*, fungal morphotypes were isolated from cuticles and cultured for the purpose of sequencing. Pure isolations from the two morphotypes were made by excising a 1 × 1 cm agar plug of fungi and subculturing it onto a new potato dextrose agar dish, sealed with parafilm to obtain a pure culture. DNA was extracted from pure cultures after 7 days of growth in an environmental chamber set to 30 °C, 60% RH, and 14:10 L:D photoperiod using the Qui*ck*-DNA Fecal/Soil Microbe Miniprep Kit (D6010, Zymo Research Corp, Irvine, CA, USA). Concentration of DNA and quality were assessed using the Take 3 Assay on a microplate reader (Gen5™, BioTek Instruments, Winooski, VT, USA) before performing the PCR.

Polymerase chain reaction (PCR) was used to amplify the internal transcribed spacer (ITS) region for both morphologically identified fungi using primer sets: ITS4 5′-TCCTCCGCTTATTGATATGC-3′ and ITS5 5′-GGAAGTAAAAGTCGTAACAAGG-3′^62^. For the morphologically identified *Fusarium* sample, the translation elongation factor 1 alpha (TEF1α) region and the RNA polymerase II subunit (RBP2) region were also amplified to facilitate species levels identification. The following primer pairs were used for TEF1α and RBP1, respectively: EF1 (5'-ATGGGTAAGGARGACAAGAC-3')/EF2 (5'-GGARGTACCAGTSATCATGTT-3') and RBP2-5F2 (5'-GGGGWGAYCAGAAGAAGGC-3')/RPB2-7cR (5'-CCCATRGCTTGYTTRCCCAT-3')^63–65^. Each reaction contained 1.0 μL extracted DNA, 1.0 µL of each primer (10 μM), 9.5 μL of nuclease free water, and 12.5 μL of master mix containing 50 units/mL of Taq DNA polymerase master mix (Hot Start Taq 2X Master Mix, Promega, Madison, WI, USA). Briefly, the PCR program consisted of 2 min of initial denaturation at 95 °C, followed by 30 cycles of 95 °C for 30 s, 55 °C for 1 min, and 72 °C for 1.5 min, and a final extension at 72 °C for 5 min (S1000 Thermal Cycler, Bio-Rad, Hercules, CA, USA). PCR products were treated with ExoSAP-it prior to sequencing following the manufacturer’s protocol (ThermoFisher Scientific, Waltham, MA, USA) Amplicons were sequenced bidirectionally on an ABI 3730XL instrument (Eurofins Scientific, Brussels, Belgium), and the resulting sequences were quality-filtered and aligned using Geneious Prime 2021.0.3 (Biomatters Ltd, Auckland, New Zealand). The consensus sequences were searched against NCBI’s nucleotide database (nt) using the BLASTn algorithm^66^. In order to circumvent taxonomic misassignments, the ITS consensus sequences were also checked against the UNITE Database using the Ribosomal Database Project Classifier algorithm^67^. The consensus sequence for the ITS sequence of *A. flavus* was submitted to GenBank under OM490684 while the ITS, TEF1α, and RBP2 sequences from *F. verticillioides* were deposited under OM460744, OM542207, and OM542208.

### Four-way olfactometer

In order to assess preference by *L. serricorne* among the stimuli, a still-air four-way olfactometer was used. This device (Sigma Scientific, LLC., Micanopy, FL, USA) consisted of a cylindrical central release glass chamber (9.0 × 12.1 cm D: H) with exit ports (2.5 × 3.5 cm D:L) at each cardinal direction connecting the central chamber to four identical glass chambers (7.0 × 11.5 cm D:H) where 10 g of each odor source was placed (Table 2). The bottom of the release arena was acid-etched to provide a surface over which insects could easily crawl. Each of the four outer chambers were spaced at a 90° angle from each other. Inert septa buffered and sealed each component. A removable glass lid with a hole was used to cover the top of the main central glass chamber to allow for airflow, but also prevent insects from exiting. A single *L. serricorne* was released into the middle of the central arena for each bioassay and given a maximum of 4 min to make a decision to choose one of the stimuli. A choice was considered to have been made when the individual traveled 2.54 cm into the adjoining 1-way exit port that branched off to the odor chamber. After every 5 replicates, the entire olfactometer was rotated 90° to control for positional effects. After every 15 replicates, the whole apparatus was washed first with methanol followed by hexane, and a new, independent set of each treatment was used, with the grain replaced. The time to decision and choice of odor treatment were recorded and an individual insect was never tested more than once. Non-responsive (NR) insects were also recorded, but excluded from the final statistical analysis. The assay was conducted in a biosafety cabinet to maintain a constant flow of air and odor. A total of n = 200 replicate individuals were tested.

### *Movement assay*

A movement assay was used to assess if fungal inoculated grain impacted close-range foraging behavior of *L. serricorne*. This was accomplished through the use of video-tracking with a network camera (GigE Network Camera, Basler AG, Germany), suspended using a pole and clamp 80 cm above the test arenas, coupled with Ethovision Software (Noldus Inc., Leesburg, VA, USA). The test arenas consisted of five petri dishes (100 × 15 mm) with 85 mm diameter filter paper adhered to the bottom of the petri dish with double sided tape, on an artist’s lightbox, each containing a single *L. serricorne* and a single treatment kernel (Table 2). Each test arena was halved vertically, with the left half designated for the control half (e.g., no kernel) and the right half for the treatment half. Embedded within each half of the arenas were 1.16 cm diameter control kernel zones or treatment kernel zones, located midway and halfway on each respective half (Supplemental Fig. 1). These were coded as hidden zones in Ethovision and the smaller treatment kernel zones were where the grain treatments were placed, consisting of a single kernel of each treatment listed in Table 2. A single *L. serricorne* was released at the midpoint in the center of each arena. Trials lasted for a period of 30 min. The Ethovison software automatically measured several variables including the total distance moved, the cumulative duration of time spent in each half/kernel zone, the frequency of entry to each half/kernel zone, and latency to entering the half/kernel zone. The location of each treatment was randomized between each replicate to control any positional bias. There was a total of n = 20 replicates per treatment. No petri dish, filter paper, or adult was ever used more than once.

### Release-recapture assay

A trapping assay was utilized to determine how microbial fungal volatiles impact the capture by *L. serricorne* in monitoring traps at a longer distance. In a large, walk-in environmental chamber (5 × 6 × 2 m, L:W:H), plastic bins (86.3 × 30.5 × 39.4 cm L:H:W) were set up to perform the experiment under constant environmental conditions (28 °C, 59% RH, 14:10 L:D). A ring of polytetrafluoroethylene (Millipore Sigma, Burlington, MA, USA) around the top and bottom of the plastic bins was added to prevent insects from climbing out of the bins. The bottom of the bins was roughened with sandpaper to provide traction. The traps consisted of commercially-available pitfall traps (Storgard, Trece, Adair, OK, USA) with two connectible pieces^68,69^, containing a central well where the treatments were added as bait. Traps were baited with treatments in Table 2 and placed in one corner inside the bins. A total of 20 adult, mixed-sex *L. serricorne* were released in the corner diagonal and on the opposite side from where the trap was placed. The positions of traps were randomized in a different corner inside the bin to reduce any positional bias between each replicate. The insects were given 24 h to respond to stimuli. Afterwards, traps were collected, and the number of insects recaptured was recorded. A total of n = 8 replicate releases were performed per treatment.

### MVOC headspace collection

To determine how microbial colonization impacted volatile emissions from grain, headspace collections were performed on fungal-inoculated, uninoculated, and UV-treated grain. The headspace volatile collection apparatus consisted of four 500-ml glass chambers, each with an air inlet and outlet. Central air was first purified, then regulated with a flow meter to 1 L/m, after which it entered the headspace chamber via PTFE tubing and was collected onto a volatile collection trap (VCT). Each glass chamber was secured during headspace volatile collection with a plastic lid and a PTFE-faced butyl septum. After each use, chambers were washed with methanol first followed by hexane inside a laminar fume hood. The VCT consisted of an angled drip-tip collection point borosilicate glass tube with a mesh (Stainless Steel #316 screen), packed with 20 mg of PoraPak-Q™ chemical absorbent held in place with a borosilicate glass wool plug, and followed by a PTFE Teflon™ compression seal, which was used to collect and concentrate MVOCs over 3 h periods from 20 g of each treatment (Table 2). Background volatiles were also collected from empty glass chambers as a negative control. Volatiles were eluted from VCTs inside the fume hood with 150 µL of dichloromethane, which was pushed through with N_2_ gas into labeled 2 mL GC vials with 150-µL glass inserts with PTFE polymer feet and magnetic seal caps. Using a microsyringe (2-μl capacity, Hamilton Co., Reno, NV, USA), 1 µL of internal standard (190.5 ng tetradecane) was added to each sample. Between each use, the syringe was washed with 2 µL of dichloromethane in triplicate to avoid cross contamination between samples. All headspace samples were sealed with Teflon tape and stored at − 13 °C until GC–MS analysis below. For reuse of VCT, traps were washed in triplicate with 700 µL of dichloromethane, which was pushed through with N_2_ gas.

### MVOC GC–MS analysis

All headspace collection sample extracts were run on an Agilent 7890B gas chromatograph (GC) equipped with an Agilent Durabond HP-5 column (30 m length, 0.250 mm diameter and 0.25 μm film thickness) with He as the carrier gas at a constant 1.2 mL/min flow and 40 cm/s velocity. This was coupled with a single-quadrupole Agilent 5997B mass spectrometer (MS). The compounds were separated by auto-injecting 1 μl of each sample under split mode into the GC–MS at room temperature (approximately 23 °C). The flow was split in a 15:1 ratio with a split flow rate of 18 ml/min. The GC program consisted of 40 °C for 1 min followed by 10 °C/min increases to 300 °C and then held for 26.5 min. After a solvent delay of 3 min, mass ranges between 50 and 550 atomic mass units were scanned. Compounds were tentatively identified by comparison of spectral data with those from the NIST 17 library and by GC retention index^70^. Using the ratio of the peak area for the internal standard to the peak area for the other compounds in the headspace, the emission rates of samples was normalized in ng of volatile per g of grain, per μl of solvent, and per h of collection were calculated.

### Statistical analysis

In order to analyze the quantitative data from the vectoring assay, the mean greyscale value was used as the response variable in a linear model. Semiochemical treatment (Table 2) was included as a fixed, explanatory variable. Residuals were inspected to validate assumptions of normality and homoscedasticity. If these assumptions were not met, log -transformed data were used, which then satisfied both assumptions. Upon a significant result of the model, Tukey HSD was used for multiple comparisons and implemented using the function *ghlt* from the package *multcomp* in R Software. R software^71^ was used for this and all other tests, with α = 0.05 except where noted.

To analyze the data from the four-way olfactometer, a Chi-squared test was performed with a Bonferroni adjustment to the *α*-threshold. The null hypothesis assumed that there was an equal probability that an insect would choose one of the four sides.

To analyze how grain and microbial volatiles affect the mobility and foraging of *L. serricorne* during video-tracking, a generalized linear model based on a Gaussian distribution with a log-link function was used. In particular, separate models were used for the distance moved, and instantaneous velocity as response variables, while the explanatory variable included the odor treatment included in each test arena (e.g. from Table 2). To detect behavioral differences in this assay, the frequency of entering each zone, cumulative duration spent in each zone, and latency to finding each zone were each analyzed using a multivariate analysis of variance (MANOVA) with the semiochemical treatments as a fixed explanatory variable. Upon a significant overall result, sequential ANOVAs were performed for each variable. For responses with significant ANOVAs, multiple comparisons were performed with Tukey HSD. Post-hoc t-tests were performed as a contrast between the two means of treatment vs. control zone and treatment vs. control kernel zone to assess differences within a semiochemical treatment with a Bonferroni-corrected alpha-threshold for multiple testing.

To analyze the data from the release-recapture assay, a generalized linear model based on a Poisson distribution and log link function was used. The response was the number of *L. serricorne* recaptured in traps for a particular stimulus. The explanatory variable included the odor treatments from Table 2. Overdispersion was not found to be a problem. Upon a significant result from the model, multiple comparisons in a generalized linear model framework were implemented using the function *ghlt* (e.g. Tukey HSD) from the package *multcomp* in R Software.

To characterize the change in headspace volatiles with inoculation of fungi in grain, raw peak areas were extracted from the gas chromatograms using MSD ChemStation v2.00 software (Agilent Technologies, Inc., Santa Clara, CA, USA). The emission rate was calculated on a ng per gram of grain weight and per hour basis by using the ratio of the peak area for tetradecane to each headspace volatile. Background volatiles found in the negative control without grain were discarded from the other samples, since these represent transient background volatiles. Pairwise Bray–Curtis similarities were calculated between each headspace sample, and non-metric multi- dimensional scaling (NMDS) was used to visualize the differences in volatile profiles among treatments. A total of n = 1000 permutations were used for the ordination procedure. Stress values for the NMDS procedure were < 0.1, indicating that good interpretation was possible. An analysis of similarity (ANOSIM) was used to determine significant differences for headspace volatile profiles among odor treatment categories. ANOSIM calculates an R statistic that can vary from 1 to − 1, with values above zero interpreted as greater dissimilarity between treatments than within and values below zero interpreted as greater dissimilarity within treatments than between treatments. A total of n = 1000 permutations were performed for the test. For all multivariate statistics, the R Package *vegan* was used^72^. To assess differences in the complexity of the headspace blends (e.g. number of compounds per treatment), pairwise chi-square tests were performed among treatments with the null hypothesis of an equal number of compounds identified from each treatment. To determine differences in overall quantitative emissions of volatiles, pairwise t-tests among means were performed using a Bonferroni-correction to the P-value.

### Ethics declaration

These studies adhered to the highest US and international standards for scientific integrity. No humans or vertebrate animals were used in these experiments. This BSL-2 research is covered and approved through the Institutional Biosafety Committee at Kansas State University under approved permit IBC#1437 through 3/17/2023.

## Supplementary Information

Supplementary Figures.

## Data Availability

The datasets generated for this study can be found in the Ag Data Commons Repository managed by the U.S. Department of Agriculture at: Ponce, Marco A.; Sierra, Petra; Maille, Jacqueline; Kim, Tania N.; Scully, Erin D.; Morrison, William R. Data from Attraction, mobility, and preference by *Lasioderma serricorne* (F.) (Coleoptera: Ptinidae) to microbially-mediated volatile emissions by two species of fungi in stored grain. Ag Data Commons. 10.15482/USDA.ADC/1528422. Accessed 2023-01-12.
