# Supplementary material for: Attraction, mobility, and preference by Lasioderma serricorne (Coleoptera: Ptinidae) to microbially-mediated volatile emissions by two species of fungi in stored grain
Source: Sci Rep. 2023 Apr 15;13:6176. doi: 10.1038/s41598-023-32973-y (PMC10105710; doi:10.1038/s41598-023-32973-y)
Supplement: Supplementary file 1 — Supplementary Figures. [file 41598_2023_32973_MOESM1_ESM.docx]

Supplementary Material

# Supplementary Figures


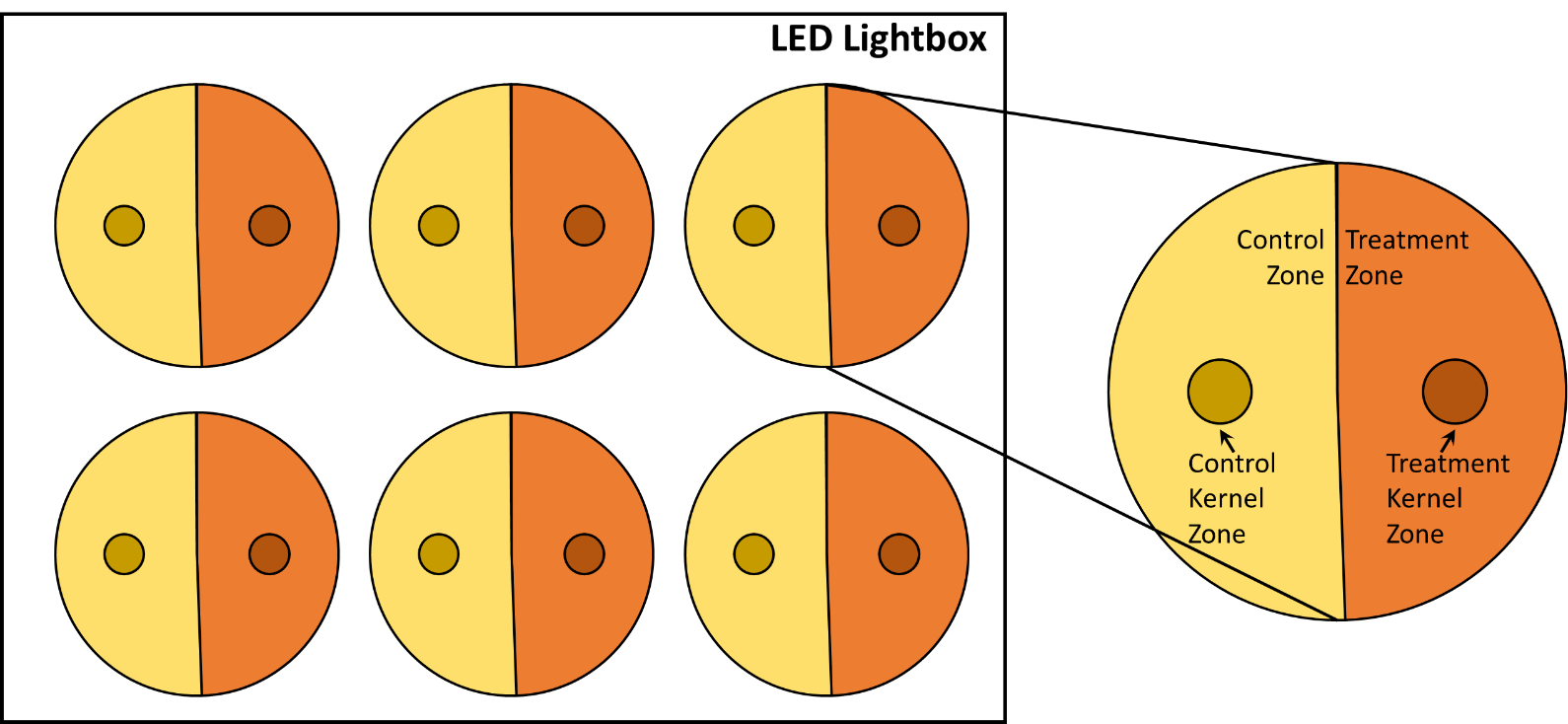


**Supplementary Figure 1.** Arena setup of Ethovision® assay showing control half (light yellow) and treatment half (light orange) of arenas and smaller hidden kernel zones where the treatment was placed (dark orange) or was left empty in the control (dark yellow). *Lasioderma serricorne* was tracked for 30 min periods in the arena from a camera suspended above the arenas.


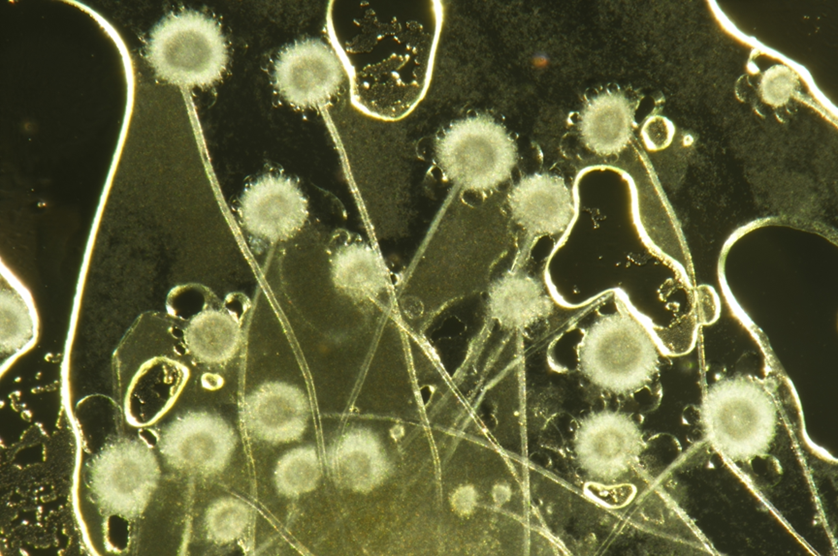


**Supplementary Figure 2.** 3D Habitus images of *Aspergillus flavus* at 10× magnification (SMZ-18, Nikon Inc., Tokyo, Japan) used to inoculate grain in assays. *A. flavus* is characterized by its green, filamentous morphology and visible spores.
